# The Impact of Self-Efficacy, Optimism, Resilience and Perceived Stress on Academic Performance and Its Subjective Evaluation: A Cross-Sectional Study

**DOI:** 10.3390/ijerph18178911

**Published:** 2021-08-24

**Authors:** Ovidiu Popa-Velea, Irina Pîrvan, Liliana Veronica Diaconescu

**Affiliations:** 1 Department of Medical Psychology, Faculty of Medicine, University of Medicine and Pharmacy “Carol Davila”, 050474 Bucharest, Romania; liliana.diaconescu@umfcd.ro; 2 Faculty of Medicine, University of Medicine and Pharmacy “Carol Davila”, 050474 Bucharest, Romania; irina.pirvan@stud.umfcd.ro

**Keywords:** self-efficacy, optimism, resilience, perceived stress, academic performance

## Abstract

This study aimed to highlight the contribution of self-efficacy, optimism, resilience and perceived stress on academic performance (GPA) at medical undergraduate students. Additionally, we investigated the relationship established between abovementioned variables, gender and position in the academic cycle and the factors associated with satisfaction regarding own academic results. 118 students (25 men, 93 women, mean age 22.863, SD = 1.763) participated in the study. They were administered the General Self-Efficacy Scale (G-SES), the Revised Life Orientation test (LOT-R), the Brief Resilience Scale (BRS) and the Perceived Stress Scale (PSS). t, Wilcoxon-Mann-Whitney, chi-square tests, and linear regressions were performed, in order to test the strength of the hypothesized associations. Data analysis demonstrates a significant association between low optimism, low resilience, high perceived stress and poor academic performance. Even though preclinical students displayed greater perceived stress, it negatively correlated to GPA only in clinical students, this indicating a possible accumulation of stress-related effects. No gender differences were met for the studied variables. Students in clinical cycle were significantly less satisfied about their own performance. These results indicate solid associations between psychosocial variables and academic performance, and differences in the subjective evaluation of own performance, suggesting key points to address, when designing interventions against academic stress.

## 1. Introduction

Academic stress is a major unwanted consequence of many undergraduate study programs across the world, significantly impairing quality of life, mental health and social integration of the affected individuals [1,2,3]. Despite consistent efforts being undertaken to address this problem, stress experienced by students continues to be widely prevalent [4,5,6,7,8] and represents a major contributor to burnout. This “occupational phenomenon” [9] can occur right from the early years of academic formation, and later durably extend onto professional life, causing many additional undesirable costs for the individual and the society [10,11,12,13,14].

Among different types of educational contexts, academic stress is especially a matter of concern in medical schools [15,16,17]. This is partially due to its multiple sources, such as the vast nature of the curriculum, possibly including “hidden contents” [18], the perceived lack of control experienced by students [19], the emotionally-charged components of the medical training [20], the substantial workload [21], concluded with frequent and difficult examinations [22] and the significant interference with other domains of life [23]. Consequently, many medical students may lose, throughout studies, the track of their initial motivation to pursue this career [24,25,26], while a gap may occur and widen between the objective evaluation of their abilities and their subjective self-evaluation [27]. This discrepancy may represent an additional reason for self-doubt and unhappiness in students pertaining to vulnerable groups [28,29].

While individual creativity, innovations and encouragement of assertiveness and autonomy could represent valuable tools for addressing these problems, organizational structures may oppositely perceive the solutions as coming from the implementation of an even stronger top-down control and more centralized decisions [30]. Regrettably, in many cases the rigidity of the frameworks in which academic medical institutions have been conceptualized and function may provide a genuine low sensitivity to the issue of academic stress, considering it “unreachable and/or unchangeable” [31]. In this particular context, personal variables can be decisive in moderating or alleviating the confrontation to academic stress. Several of these variables have been quoted in literature to have particular importance:

- Self-efficacy, defined as the ability “to set higher goals, commit to challenges that are more difficult and strive to meet those goals” [32], is often considered “the most important predictor of change in behavior” [33]. It represents a significant resource, able to substantially predict academic success, for example the first-year college GPA, the number of accumulated credits and college retention after the first year [34]. There is a strong correlation between self-efficacy and students’ adjustment to university life, with those with a high level of self-efficacy perceiving transition as a challenge rather than a threat [35]. Self-efficacy correlates with effort regulation, deep processing strategies and goal orientation [36]. It is less clear however to what extent self-efficacy remains associated throughout studies with feelings of personal satisfaction and accomplishment about personal academic results.
- Resilience is conceptualized as “the ability to maintain or regain mental health, despite experiencing adversity” [37] and has been reported to predict academic performance, especially through the dimensions of hardiness and resourcefulness [38]. This effect is not exclusively direct, but it could be mediated by additional factors, such as the increased ability of resilient students to use social support networks [39] or to perform healthy behaviors [40].
- Optimism, within certain ranges, has generally a positive correlation with academic achievement, and especially with its self-perception [41]. These relationships are partially explained by the way optimistic students both think and subjectively relate to specific stressful events, such as intensive learning or confrontation to exams. In the case of medical and allied health undergraduates, literature findings show that they are significantly more active and able to efficiently use adaptive coping strategies, such as problem-solving and social support [6,42,43]. In addition, they are more relaxed and involved in their tasks, experience a higher sense of competence and invest more effort to achieve their goals [44,45,46]. On a long run, they may feel encouraged for novel, varied and exploratory thoughts and actions, potentially leading to new skills and resources [47,48], which could further feed academic performance. Still, one should notice that the magnitude of these associations may differ, according to gender and personality characteristics [49], and may reach a plateau in above-average optimism ranges [50], or even reverse, in students with extreme low or high optimism levels [51].
- Perceived stress has been described as being more prevalent in academic contexts [6,52] and being linked to poor academic outcomes [53] and a higher risk of burnout [20,54,55,56]. The negative relationship between stress and academic performance can be mediated by psychiatric or psychosomatic symptoms, or by disturbances of the biological rhythms. In the latter category, a particular importance is attributed to sleep disorders, as they can impair on a long run the consolidation of new notions and the recollection of the already-learned information [57,58]. In students affected by these conditions, poor academic performance can be not only an effect, but also a cause for further disability, thus leading to a vicious circle [59]. It is remarkable to notice that perceived academic stress seems to impact even the infrastructural neuronal level, disrupting plasticity [60], possibly via high secretion of cortisol [61] and malfunctioning of the limbic system [62]. Consequently, abilities which are critical for the academic performance, such as the acquiring and memorizing of new information, may be durably impaired [63,64].

Despite these undeniable associations, the link between perceived stress and academic performance can be a complex one, with literature reports about moderate stress actually being a stimulus for students to enhance their performance [65,66]. For these reasons, the study of academic stress dynamics, especially in long-term studies, such as Medicine, remains a challenging study topic.

Even though the abovementioned variables seem to significantly influence academic performance and its subjective evaluation, little is known about their comparative importance in exerting this action. Furthermore, their influence can be gender-related, or sensitive to the content of the academic curriculum, with a potential significant change occurring at the transition from preclinical to clinical instruction. The latter corresponds to a shift from mostly theoretical notions to the exercise of practical skills and confrontation to emotionally-charged clinical situations. This can bring feelings of frustration, incompetence and dissatisfaction with the way individual performance is assessed, which can be counterproductive and represent an additional source of demotivation and burnout.

In this sense, our study aims to clarify the individual role and the comparative importance of self-efficacy, resilience, optimism and perceived stress on academic performance in Medicine undergraduates. Additionally, we considered in our analyses the differences of psychological variables and Grade Point Average (GPA) attributable to gender and the position in the academic cycle, and the subjective evaluation of own academic performance. These data could be particularly important in Romania, a country where the prevalence of stress-related disorders and burnout in the academic settings is rising and where the support systems for students facing academic difficulties are scarce and not yet effective enough [31]. Beyond our explorative intention, which fills a gap in current knowledge of this research area, these data could be important for educational counselors and psychologists in addressing specific psychological variables, or in considering care strategies, tailored to specific contexts in which interventions are needed.

## 2. Materials and Methods

### 2.1. Design

The design of the study was cross-sectional, with a single administration of a series of standardized psychometric instruments.

### 2.2. Participants

Participants were undergraduate students undergoing their training at the University of Medicine and Pharmacy “Carol Davila” Bucharest, Romania (UMFCD). This university is the largest medical school in Romania, gathering students from all regions of the country. 118 students (25 men, 93 women; mean age = 22.864, SD = 1.763, range 18–27) agreed to participate in the study. All participants met the inclusion criteria, set as being at least 18 years of age and having the status of current undergraduate students in the abovementioned institution. Exclusion criteria were represented by current self-reported somatic or psychiatric morbidity, cognitive deficits or any other impairments which would render the understanding and completion of the study questionnaires difficult, and lack of completion of one or more study instruments. 78.81% of participants were women, which is consistent with the high proportion of female enrollment in Romanian medical schools. The sex ratio between men and women in our sample (25/93 = 1:3.72), although higher from the sex ratio of all students enrolled in the university (1:2.38) was not significantly different (χ^2^ = 3.106, *p* < 0.08, *ns*).

### 2.3. Procedure

Data were gathered in March–May 2021, through the administering of an online set of questions, containing the study instruments. Before taking part in this research, all participants received a brief explanatory statement about the study and completed informed consent forms. The study was run in accordance with the World Medical Association Declaration of Helsinki and was approved by the UMFCD Institutional Review Board (no. 7873/2021). A researcher (IP) was available by phone or email, in case there were questions related to the process of filling the questionnaires. All responses were processed anonymously and a numerical code was assigned for each participant. The collected data were accessible exclusively to study researchers (OPV, IP, LVD). Regular didactic staff had no access to the distribution, collection or interpretation of questionnaires. The interpretation of the questionnaires was performed independently by two researchers (IP, LVD) and cross-checked for congruence afterwards. Final results were included in a SPSS 21 (SPSS^®^ Inc., Chicago, IL, USA) database.

### 2.4. Instruments

All participants received four questionnaires—General Self-Efficacy Scale (G-SES), The Revised Life Orientation test (LOT-R), The Brief Resilience Scale (BRS) and the Perceived Stress Scale (PSS), which were chosen based on the existing literature data supporting their adequacy to the aims of the study:

1. The General Self-Efficacy Scale (G-SES) [67] comprises 10 items and is used to assess a person’s beliefs about their own ability to cope with difficulties encountered during solving tasks. Example of areas investigated by the questionnaire include ability to solve difficult problems, consistency in following one’s goals or the propensity to recourse to own resourcefulness and positive emotions. The answering options vary from 1 to 4, where 1 = “completely untrue”, and 4 = “perfectly true”. The total scores may range between 10 and 40. The scale has been reported to display good psychometric properties, with Cronbach’s alpha index values between 0.79 and 0.93 [68,69];
2. The Revised Life Orientation test (LOT-R) [70] comprises 10 items and evaluates optimism versus pessimism. Items investigate aspects such as enjoyment in social life, resistance to bad or unforeseen circumstances or ability to relax. The answers are provided on a Likert scale from 0 to 4, where 0 = “strongly disagree” and 4 = “strongly agree”. The scale has good construct validity and reliability in both clinical and non-clinical samples, including students, with values of Cronbach’s alpha between 0.72 and 0.78 [71,72].
3. The Brief Resilience Scale [73] assesses a person’s ability to recover after a stressful period. Example of behaviors investigated by this test include the ability to bounce back in negative circumstances or to recover after facing acute stress. It comprises 6 items and a 5-steps Likert response scale, where 1 represents “total disagreement” and 5 “total agreement”. The scale has good properties in assessing resilience in undergraduate students, with Cronbach’s alpha above 0.70 and good construct validity [74].
4. The Perceived Stress Scale (PSS) [75] is a 14-item self-report instrument, designed to measure the degree to which situations in one’s life are appraised as stressful. Items investigate aspects such as the emotional reaction to negative life situations, the degree of perceived control over sources of stress or the confidence in handling one’s problems. Each item is rated using a 5-point Likert type scale, with a total score ranging between 0 and 56. This psychometric tool has been empirically validated with populations of college students [76] and depicts convergent validity, indicated by its solid relationships with scales measuring depression and somatic symptoms [75].

In addition to these four psychometric instruments, the participants provided information about their age at the time of the testing, the academic year in which they were enrolled and a subjective assessment about their perceived satisfaction concerning the own academic performance in previous years (expressed as a dichotomic answer yes/no).

The distinct variable to which the results of the psychometric tests were referred to was the Grade Point Average (GPA). In Romanian medical universities, for each distinct class (both preclinical or clinical), an objective grade is offered, ranging from 1 (worst performance) to 10 (best performance). In this study, the student’s GPA was calculated at the end of an academic year as a weighted mean of grades, according to the number of credits assigned to each class (different classes can have a different number of credits, so their load in the calculation of the GPA can differ).

### 2.5. Data Analysis

The analysis included firstly a descriptive level and an assessment (via Shapiro Wilk tests) of the position of the study variables in the normality-abnormality continuum. According to this outcome, a series of *t*-tests for independent samples and Wilcoxon-Mann-Whitney tests were run to assess differences in self-efficacy, perceived stress and, respectively, resilience, optimism and academic performance, which were attributable to gender and position in the academic cycle (preclinical or clinical). Similarly, chi-square and Student or Wilcoxon-Mann-Whitney tests were performed to document the differences in terms of satisfaction regarding own performance, in relationship to self-efficacy, optimism, resilience, perceived stress and GPA. Linear regression was performed to establish the comparative weight of GPA predictors, by grouping independent variables into demographical ones (age, gender, position in the academic cycle) and psychological ones (self-efficacy, optimism, resilience, perceived stress). This last analysis was separately run for students in the upper and the lower GPA quartile. Throughout statistical analyses, missing data were handled through list wise deletion. Assumptions of data normality and linearity were confirmed using histograms and partial plots. The independence of errors was checked through the Durbin-Watson test. For all calculations, the threshold of statistical significance was *p* < 0.05.

## 3. Results

### 3.1. Study Variables

A synthesis of the mean, standard deviation, minimum and maximum values of independent and dependent variables, as well as of their distribution, in terms of normality, is depicted in Table 1.

The normal distribution was ensured for self-efficacy and perceived stress and not met for optimism, resilience and GPA. Consequently, two different types of statistical tests were used for further comparisons concerning these two distinct categories of variables (3.3 and 3.4).

### 3.2. Determinants of Academic Performance (GPA)

A series of multivariate regression analyses aimed to investigate the distinct association between demographical and psychological variables, and academic performance (measured through the students’ GPA).

In a first step, these analyses compared students with poor academic performance (in the lower quartile, i.e., GPA below 8.62) to students with high academic performance (in the upper quartile, i.e., GPA above 9.64). The investigated demographical variables were gender and age, while the psychological variables were self-efficacy, optimism, resilience, perceived stress and satisfaction regarding own academic performance. These results are displayed in Table 2 and Table 3.

For this category of students, the second depicted model was statistically significant (F = 2.919, *p* < 0.017). The GPA predictors for this student category were optimism (t = 2.819, *p* < 0.033), resilience (t = 2.727, *p* < 0.039) and perceived stress (t = −2.575, *p* < 0.018).

None of the models proved to be statistically significant (F = 1.323, *p* < 0.284 at model 1; F = 0.815, *p* < 0.585 at model 2).

In the second step, we realized a similar analysis of the determinants of academic performance, by taking into consideration the position in the study cycle (preclinical and clinical). These results are depicted in Table 4 and Table 5.

None of the two models was statistically significant (F = 1.712, *p* < 0.209 at model 1; F = 0.904, *p* < 0.532 at model 2).

For clinical students, although none of the models was statistically significant, a certain importance of perceived stress was evident in model 2 (t = −2.641, *p* < 0.010).

### 3.3. Analysis of Variables, by Gender and Position in the Academic Cycle

This analysis was conducted via Student tests for independent samples (in the case of Self-efficacy and Perceived stress) and via Wilcoxon–Mann–Whitney two-sample rank-sum test (in the case of Optimism, Resilience and GPA) (Table 6 and Table 7).

No significant differences were met, according to the participants’ gender (*p* < 0.220–0.976).

The only significant difference between clinical and preclinical students consists in the amount of perceived stress, higher in preclinical students (*p* < 0.028).

### 3.4. Subjective Evaluation of Own Academic Performance

Table 8 depicts the inventory of differences met between students who were dissatisfied and, respectively, satisfied with their academic performance (GPA).

The significant satisfaction differences were related to the position in the academic cycle (students in clinical academic years were significantly less satisfied; *p* < 0.019) and to the GPA itself (dissatisfied students had lower GPAs; *p* < 0.012).

## 4. Discussion

This study aimed to clarify the individual role and the comparative importance of several psychological variables (self-efficacy, resilience, optimism and perceived stress) on academic performance. Additionally, we investigated the possible associations between these variables and gender and position in the academic cycle. Finally, we examined the factors associated with dissatisfaction, respectively, satisfaction in respect to own academic results.

Regarding the first objective, a substantial percentage (34.1%) of GPA variance was predicted, in the case of low performance students, by psychological variables, specifically low optimism (*p* < 0.033), low resilience (*p* < 0.039) and high perceived stress (*p* < 0.018). This is in concordance with existing literature data, which distinctly point out each of these factors as substantially affecting academic performance. Specifically, students with low scores in optimism typically display a generalized expectancy about bad outcomes in life [77], which might diminish their expectations about academic performance, subsequently influencing their motivation and behavior at school and exposing them to burnout [78]. Inversely, optimistic students may use more often problem-focused strategies in controllable situations related to academic challenges [79], emphasize the positive aspects of the encountered difficulties and be more confident when dealing with them [80]. On their turn, students with low resilience may be deprived by the buffer effect of resilience on the relationship between adjustment problems and academic performance [81]. Lastly, perceived stress has been consistently reported as a distinct variable able to negatively influence academic achievement, via multiple, sometimes complimentary mechanisms [82,83,84].

Interestingly, in what concerns high academic performance (expressed as a GPA placed in the upper quartile), this was predicted in a much lesser amount (namely 4.8% of the GPAs variance) by the considered psychological variables. This could be explained by the intervention, in high performing students, of additional factors that can shape performance, such as learned resourcefulness [85], conscientiousness [49], sense of coherence [86] or self-esteem [87], which were not explored in this study.

In terms of the second study objective, we were able to identify statistically significant differences between preclinical and clinical students. Specifically, the amount of perceived stress in preclinical students was significantly higher than in clinical students (*p* < 0.028). However, GPA and perceived stress correlated negatively only in clinical students (*p* < 0.004), suggesting a possible cumulative effect of the academic stress perceived in preclinical years. This stress load can evolve later in the academic cycle into burnout [88], making the identification of high perceived stress in early years of education a priority for school counselors and tutors, as well as for students themselves.

Remarkably, in our sample gender differences have not been proved to be statistically significant. Self-efficacy, optimism, resilience, perceived stress and GPA were similar in men and women, a finding that could reflect a symmetry in what concerns the gender-wise development of these parameters. Still, these findings could be equally explained, at least partially, by the low number of male subjects having participated in the study.

In what concerns the third study objective, the subjective evaluation of own academic performance depended on the position in the academic cycle (with clinical students less satisfied, *p* < 0.019), and on the GPA itself (*p* < 0.012). These results argue in favor of higher demands put by the students on themselves in the clinical years of their academic formation, possibly in view of their forthcoming career as physicians. This hypothesis is strengthened by the large number (*N* = 41) of 6th year students dissatisfied with their academic performance. Overall, this high percentage of dissatisfied students can represent a warning sign, from the perspective of the inner motivation needed for a successful entering on the medical job market. In addition, from the perspective of burnout risk, this could represent a potential unfavorable circumstance, given the presence of “Low personal accomplishment” as a distinct component of this condition.

This study has a number of limitations, among which the most important are its cross-sectional design, the limited number of respondents, their self-selection, the sex ratio skewed towards women and the comparatively small sample of preclinical students. The exclusion criteria may have removed from the study potentially interesting participants, namely those with history of stress-related disorders. The dissatisfaction about academic performance may have remained unreported, especially in those students who engage in health risk behaviors (e.g., use of energy drinks, psychostimulants, cannabis), to increase their academic performance and to better cope with academic stress [89,90]. These flaws could be remedied through the design of future multicenter prospective studies, including a larger diversity of students and investigating a larger number of psychosocial variables able to influence academic performance.

## 5. Conclusions

The results of this study point out the associations between low resilience, low optimism, high perceived stress and poor academic performance in medical undergraduates. In contrast, high academic performance has not been predicted by these variables, creating the possibility for the involvement of additional psychosocial factors that need to be addressed in further research. Perceived stress has been higher in preclinical students, however academic performance and perceived stress correlated negatively in clinical students only. This suggests a cumulative effect of academic stress in preclinical years that impacts academic life in the clinical cycle. The risks brought by this increased stress load is supplemented by the dissatisfaction regarding own academic performance, which was higher in clinical students, possibly echoing the insecurity about their capacity to properly handle clinical duties and responsibilities.

As a whole, our study represents an argument for the necessity to construct and apply early in medical education preventive and therapeutic programs against academic stress and promoting resilience and positive emotions. These strategies can be taught as part of the medical curriculum, or could be implemented with the help of counselors and tutors. In terms of cost-benefit ratio, the recourse to these measures could be much more efficient than intervening on already-existent burnout, and could ensure a long-term preservation of mental health in both medical students and physicians.

## Figures and Tables

**Table 1:** Scores of the study variables.

Variable	Mean	Standard Deviation	Minimum Value	Maximum Value	Normality Check of Distribution		
					Statistic	df	Significance
Self-efficacy	29.203	5.296	16	40	0.984 *	118	0.167
Optimism	19.771	5.326	9	29	0.965 *	118	0.003
Resilience	18.118	1.872	13	25	0.952 *	118	0.001
Perceived stress	30.440	3.556	23	38	0.981 *	118	0.090
GPA	9.104	0.713	7.30	10	0.914 *	118	0.001

* Shapiro-Wilk test, GPA = Grade Point Average.

**Table 2:** Determinants of academic performance for students in the lower GPA quartile. (GPA < 8.32) (*N* = 22) (linear regression).

Model	R	R^2^	Adj.R^2^	SEE	Coefficients			t	*p*
					Variable	B	Beta		
1	0.215	0.046	−0.027	0.358	(constant)	8.087		10.715	0.000
					Gender	0.169	0.218	1.124	0.271
					Age	−0.004	−0.024	−0.123	0.903
2	0.725	0.531	0.341	0.318	(constant)	10.058		8.790	0.000
					Gender	0.179	0.023	1.299	0.208
					Age	0.042	0.024	1.103	0.283
					Self-efficacy	0.007	0.117	0.534	0.599
					Optimism	0.029	0.475	2.819	0.033
					Resilience	0.047	0.307	2.727	0.039
					Perceived stress	−0.053	−0.569	−2.575	0.018
					Satisfaction regarding own academic performance	−0.023	−0.307	−1.411	0.173

Model 1 predictors: (constant), Gender, Age. Model 2 predictors: (Constant), Gender, Age, Self-efficacy, Optimism, Resilience, Perceived stress, Satisfaction regarding own academic performance. Adj. = adjusted; SEE = standard error of the estimate.

**Table 3:** Determinants of academic performance for students in the upper GPA quartile. (GPA > 9.64) (*N* = 29) (linear regression).

Model	R	R^2^	Adj.R^2^	SEE	Coefficients			t	*p*
					Variable	B	Beta		
1	0.304	0.092	0.023	0.123	(constant)	9.473		25.910	0.000
					Gender	−0.073	−0.256	−1.353	0.188
					Age	0.017	0.208	1.100	0.281
2	0.462	0.214	−0.048	0.127	(constant)	9.695		11.730	0.000
					Gender	−0.095	−0.333	−1.465	0.158
					Age	0.027	0.323	0.868	0.395
					Self-efficacy	0.005	0.187	0.817	0.423
					Optimism	−0.005	−0.197	−0.833	0.414
					Resilience	−0.016	−0.186	−0.775	0.447
					Perceived stress	−0.004	−0.130	−0.582	0.567
					Satisfaction regarding own academic performance	−0.088	−0.220	−0.602	0.554

Model 1 predictors: (constant), Gender, Age. Model 2 predictors: (Constant), Gender, Age, Self-efficacy, Optimism, Resilience, Perceived stress, Satisfaction regarding own academic performance. Adj. = adjusted; SEE = standard error of the estimate.

**Table 4:** Determinants of academic performance for preclinical students. (*N* = 21) (linear regression).

Model	R	R^2^	Adj.R^2^	SEE	Coefficients			t	*p*
					Variable	B	Beta		
1	0.400	0.160	0.067	0.680	(constant)	13.414		4.685	0.000
					Gender	0.411	0.254	1.157	0.263
					Age	−0.233	−0.360	−1.638	0.119
2	0.572	0.327	−0.035	0.716	(constant)	10.019		2.075	0.058
					Gender	0.645	0.400	1.549	0.145
					Age	−0.229	0.174	−1.318	0.210
					Self-efficacy	−0.026	−0.201	−0.769	0.455
					Optimism	0.015	0.110	0.403	0.693
					Resilience	0.090	0.349	1.470	0.165
					Perceived stress	0.067	0.247	0.919	0.375
					Satisfaction regarding own academic performance	−0.082	−0.054	−0.202	0.843

Model 1 predictors: (constant), Gender, Age. Model 2 predictors: (Constant), Gender, Age, Self-efficacy, Optimism, Resilience, Perceived stress, Satisfaction regarding own academic performance. Adj. = adjusted; SEE = standard error of the estimate.

**Table 5:** Determinants of academic performance for clinical students. (*N* = 97) (linear regression).

Model	R	R^2^	Adj.R^2^	SEE	Coefficients			t	*p*
					Variable	B	Beta		
1	0.266	0.071	0.051	0.683	(constant)	6.006		4.777	0.000
					Gender	0.037	0.124	1.544	0.053
					Age	−0.186	−0.108	−1.079	0.283
2	0.436	0.190	0.127	0.655	(constant)	8.012		4.889	0.000
					Gender	−0.220	0.168	−1.309	0.194
					Age	−0.013	−0.120	−1.538	0.056
					Self-efficacy	0.003	0.025	0.209	0.835
					Optimism	0.001	0.009	0.075	0.940
					Resilience	0.022	0.052	0.530	0.598
					Perceived stress	−0.054	−0.282	−2.641	0.010
					Satisfaction regarding own academic performance	−0.279	−0.188	−1.947	0.055

Model 1 predictors: (constant), Gender, Age. Model 2 predictors: (Constant), Gender, Age, Self-efficacy, Optimism, Resilience, Perceived stress, Satisfaction regarding own academic performance. Adj. = adjusted; SEE = standard error of the estimate.

**Table 6:** Analysis of variables by gender.

Variables		Gender	
		Male	Female
		*N* = 25	*N* = 93
Self-efficacy	Mean	30.360	28.892
	Standard deviation	5.106	5.329
	Student test for independent samples	−1.233	
	df	116	
	*p*	0.220	
Optimism	Mean rank	59.680	59.451
	Sum of ranks	1492.000	5529.000
	Wilcoxon–Mann–Whitney two-sample rank-sum test	1158.000	
	Z	−0.03	
	*p*	0.976	
Resilience	Mean rank	59.720	59.440
	Sum of ranks	1493.000	5528.000
	Wilcoxon–Mann–Whitney two-sample rank-sum test	1157.000	
	Z	−0.037	
	*p*	0.971	
Perceived stress	Mean	30.280	30.483
	Standard deviation	3.434	3.604
	Student test for independent samples	0.254	
	df	116	
	*p*	0.800	
GPA	Mean rank	57.700	59.983
	Sum of ranks	1442.500	5578.500
	Wilcoxon–Mann–Whitney two-sample rank-sum test	1117.500	
	Z	−0.296	
	*p*	0.767	

**Table 7:** Analysis of variables by position in the academic cycle.

Variables		Position in the Academic Cycle	
		Preclinical	Clinical
		*N* = 21	*N* = 97
Self-efficacy	Mean	30.047	29.020
	Standard deviation	5.352	5.293
	Student test for independent samples	0.805	
	df	116	
	*p*	0.423	
Optimism	Mean rank	61.785	59.005
	Sum of ranks	1297.500	5723.500
	Wilcoxon–Mann–Whitney two-sample rank-sum test	970.500	
	Z	−0.338	
	*p*	0.735	
Resilience	Mean rank	61.619	59.041
	Sum of ranks	1294.000	5727.000
	Wilcoxon–Mann–Whitney two-sample rank-sum test	974.000	
	Z	−0.319	
	*p*	0.750	
Perceived stress	Mean	31.714	30.164
	Standard deviation	2.591	3.685
	Student test for independent samples	2.285	
	df	116	
	*p*	0.028*	
GPA	Mean rank	48.976	58.644
	Sum of ranks	944.500	5076.500
	Wilcoxon–Mann–Whitney two-sample rank-sum test	913.500	
	Z	−1.947	
	*p*	0.062	

**Table 8:** Determinants of perceived academic performance.

Variables		Dissatisfaction Regarding Own Academic Performance (*N* = 80)	Satisfaction Regarding Own Academic Performance (*N* = 38)	Differences between Groups	
				Statistical Test	*p*
A. Demographical variables					
Gender	Male (*N*)	15	10	χ^2^ = 0.883 (df = 1)	0.347
	Female (*N*)	65	28		
Age	Mean score	22.875	22.842	t = 0.094 (df = 116)	0.925
	Standard deviation	1.641	2.021		
	Min.-Max. score	19–27	18–27		
Position in the academic cycle	First year (*N*)	5	2	χ^2^ = 13.546 (df = 5)	0.019
	Second year (*N*)	10	4		
	Third year (*N*)	7	4		
	Fourth year (*N*)	10	4		
	Fifth year (*N*)	7	8		
	Sixth year (*N*)	41	16		
B. Psychological variables					
Self-efficacy	Mean	29.562	29.447	t = 1.069 (df = 116)	0.287
	Standard deviation	5.395	5.066		
	Min.-Max. score	16–40	18–38		
Optimism	Mean	20.087	19.105	Wilcoxon-Mann- Whitney test = 1188.500 (Z = −1.137)	0.255
	Standard deviation	5.127	5.737		
	Min.-Max. score	9–29	10–29		
Resilience	Mean	18.162	18.026	Wilcoxon-Mann- Whitney test = 1277.500 (Z = −0.608)	0.443
	Standard deviation	1.977	1.651		
	Min.-Max. score	13–25	16–23		
Perceived stress	Mean	30.475	30.368	t = 0.152 (df = 116)	0.880
	Standard deviation	3.482	3.752		
	Min.-Max. score	25–38	23–38		
Grade point average	Mean	9.020	9.280	Wilcoxon-Mann- Whitney test = 960.000 (Z = −2.519)	0.012
	Standard deviation	0.709	0.697		
	Min.-Max. score	7.40–10.00	7.30–10.00		

## Data Availability

The dataset presented in this study is available on reasonable request from the corresponding author.
